# Inhibition of cell proliferation by Tas of foamy viruses through cell cycle arrest or apoptosis underlines the different mechanisms of virus–host interactions

**DOI:** 10.1080/21505594.2022.2029329

**Published:** 2022-02-08

**Authors:** Wei Jie, Zhang Rui-Fen, Hu Zhong-Xiang, Wu Yan, Liu Wei-Na, Ma Yong-Ping, Song Jing, Chen Jing-Yi, Liu Wan-Hong, He Xiao-Hua, Li Zhi, Sun Yan

**Affiliations:** a College of Life Sciences, Shaanxi Normal University, Xi’an, P. R. China; b School of Medicine, Wuhan University, Wuhan, P. R. China

**Keywords:** Cell proliferation, foamy virus, transactivator, apoptosis, Foxo3, cell cycle arrest, PPM1E

## Abstract

Foamy viruses belong to the *Spumaretrovirinae* subfamily member of the *Retroviridae* family and produce nonpathogenic infection to hosts in the natural conditions. However, infections of foamy viruses can dramatically cause severe cytopathic effects *in vitro*. To date, the exact molecular mechanism has remained unclear which implied the tremendous importance of virus-host cell immune reactions. In this study, we found that the transactivator Tas in two foamy viruses isolated from Old World Monkey (OWM) induced obvious inhibition of cell proliferation via the upregulation of Foxo3a expression. It was mediated by the generation of ROS and the initiation of ER stress, and ultimately, the mitochondrial apoptosis pathway was triggered. Notably, PFV Tas contributed to the accumulation of G0/G1 phase cycle arrest induced by the activation of the p53 signaling pathway and the nuclear transportation of HDAC4 via upregulating PPM1E expression. Together, these results demonstrated the different survival strategies by which foamy virus can hijack host cell cytokines and regulate virus-host cell interactions.

## Introduction

Foamy viruses (FVs) are classified into the only genus in the subfamily *Spumaretrovirinae* of the *Retroviridae* family [1]. They infect a broad spectrum of hosts and have coevolved with their hosts for millions of years [2]. And FVs are also highly prevalent among simians, such as orangutans, macaques, and African green monkeys. Prototype foamy virus (PFV), the consequence of cross-species transmission from chimpanzees to humans through exposure to nonhuman primates (NHPs) [2], appears to have no pathogenicity *in vivo* but exerted a severe cytopathic effect *in vitro* [3]. The replication of foamy virus was reported to be limited to a relatively expendable, superficial cell type, and this special characteristic might be accountable for lower disease association of foamy virus infection *in vivo* [4], but the exact molecular mechanism of the highly cytopathic infection of PFV *in vitro* remains unknown. Recently, the early events in viral infection were discovered to induce sophisticated molecular pathways, which lead to the dysfunction of the host cell defense system. Thereafter, the destruction of host cells ensures virus survival, replication, and proliferation. Tas, as an important transactivator of foamy virus, regulates the early events of foamy virus infection by initiating the transcription of structural proteins and dominating viral replication [5]. Its interaction with cytokines, such as PML [6], Pirh2 [7], IFP35 [8], Nmi [9] and RelB [10], was verified, and these interactions might regulate host cell defenses and contribute to virus-host cell interactions.

The arsenal of defense mechanisms that trigger the premature death of infected host cells has been identified as a powerful mechanism to curtail viral spread [11]. Mitochondrial apoptosis pathway is mediated by virous apoptosis-related factors (Bax, Bak and Bcl-2, Bcl-XL), which are induced by changes in mitochondrial surface charge and the release of cytochrome C into the cytoplasm [12,13]. Moreover, as an important member of the forkhead protein family, the cytokines Foxo3a is reported to activate the function of detoxification enzymes, such as superoxide dismutase 2 (SOD2), catalase (CAT), or Sestrins. Subsequently, they contributes to changes of outer mitochondrial membrane permeability and triggers the mitochondrial apoptosis pathways [14–18]. Another typical signaling pathway, endoplasmic reticulum stress (ER stress), mainly regulated by three factors: endoplasmic reticulum kinase (PERK), activated transcription factor 6 (ATF6), and inositol demand protein 1 (IRE1) [19,20], is also suggested to activate mitochondrial apoptosis by inducing a dramatic increase in Chop expression [21]. In addition, ROS, as a highly reactive molecules produced by mitochondria, is reported to enhance ER stress and cause cell apoptosis ultimately [22–24]. A recent study showed that viral infection influences the host cell cycle through the dynamic phosphorylation regulation on cell cytokine [25]. Protein phosphorylation regulation exerts great importance in the process of cell proliferation, development, and signal transduction and is also critical for foamy virus infection [26]. PPM1E, a Ca2+/calmodulin-dependent protein kinase phosphatase, reportedly regulates the dephosphorylation of substrates [27–29]. CaMKs (Ca2+/calmodulin-dependent protein kinases) [30] and AMPK [31,32] has been reported to be dephosphorylated by PPM1E, leading to the nuclear-cytoplasmic trafficking of HDAC4, which is vital in regulating cell cycle arrest [33–35]. Furthermore, the p53 signaling pathway may regulate cell proliferation by upregulating the downstream factor p21 and inhibiting the effect of CDK2 on the G1 phase check point [36].

In our previous studies, phylogenetic and motif conservation analyses demonstrated that the Tas of SFVagm and SFVora, simian foamy viruses from Old World monkeys, are much more similar to PFV Tas (unpublished data). Therefore, the effect of SFVagm, SFVora, and PFV Tas on cell proliferation was determined in this study to explore the host cell self-protective reaction against infection with foamy virus and to further investigate the different viral survival strategies as specific cell signaling pathways induced by early infectious events after different virus infection.

The results revealed a remarkable phenomenon in which Tas of SFVagm and SFVora induces cell proliferation via cell apoptosis, while PFV Tas exhibited significant G0/G1 phase arrest. Furthermore, to confirm the regulation on the signaling pathways, which played a prominent part in host self-protection and viral survival strategies, a variety of classical experiments were performed to detect significant changes among typical proteins and ultimately verify the exact molecular mechanisms. These findings provide a foundation for understanding the severe cytotoxicity induced by Tas of foamy virus and shed light on virus-host cell interactions.

## Materials and methods

### Cell culture and construction of stable cell lines

HeLa and 293TN cells were maintained in DMEM (12,100, Solarbio, China) supplemented with 10% FBS (CP17-1616, Capricorn, Germany) and 1% penicillin/streptomycin (P1400, Solarbio, China) in 5% CO_2_ at 37°C. For stable 3˟Flag-Tas-overexpressing cell-line establishment, a Lenti-X™ Tet-On® 3 G Inducible Expression System (Clontech® Laboratories) was used following the manufacturer’s instructions. For knocking out PPM1E, sgRNA (GGGCCAAGCTGTTGAACTA) using the Lentiviral Crispr Toolbox released by ZhangLab was subcloned into LentiCRISPRv2 and then cotransfected with pMD2.0 and psPAX2. Seventy-two hours after transfection, the supernatant of lentiviruses was collected and used to infect HeLa cells for 48 h. Finally, the obtained stable cell lines in which PPM1E was knocked out were selected by puromycin resistance screening in 1 µg/ml for 7 days.

### MTT assay

The cells with inducible gene expression were inoculated for successful attachment. After the rinse with PBS, MTT was added and incubated subsequently at 37°C for 4 h. Then, DMSO was added and vibrated gently to make the complex dissolve sufficiently. Finally, a microplate analyzer (Thermo Scientific, USA) was used to measure the absorbance of crystalline complex. For the analysis of cell proliferation, it was expressed as the average optical density at 490 nm ± SEM (n = 3).

### Cell cycle analysis

Cell cycle analysis was conducted by Cell Cycle Detection Kit (KGA511, KeyGEN BioTECH, China) following the manufacturer’s instructions. In a nutshell, cells were fixed with 70% ethanol at 4°C for 2 hours and resuspended in propidium iodide (PI) solution supplemented with 10% RNase A at 25°C in the dark for 30 min. Thereafter, 10,000 events per sample were analyzed to tested the cell apoptosis by a flow cytometer (CytoFLEX, Beckman Coulter). The histogram was determined by ModFit LT 5.0 software (Verity Software House, Topsham, ME) and presented as proportion of the cells in the G0/G1, S, and G2/M phases.

### Apoptosis assay by flow cytometry (FACS)

Apoptosis assays were conducted by an Annexin V-APC/PI apoptosis detection kit (Biosciences) following the manufacturer’s instructions. In a nutshell, cells were collected and stained with 5 µl of Annexin-V APC and 5 µl PI at 25°C in the dark for 15 min. Thereafter, 10,000 events per sample were analyzed to tested apoptosis by a flow cytometer (CytoFLEX, Beckman Coulter).

### Quantitative PCR

PPM1E mRNA expression levels upregulated by Tas were assessed by RT-PCR on a Bio-Rad CFX96 Real-Time System. After HeLa cells were transiently transfected with Tas, total RNA was isolated using RNAiso (Takara, Japan), and this RNA was reverse-transcribed to first strand cDNA by M-MLV (RNseH-) reverse transcriptase (Takara, Japan). Gene expression analysis was conducted by TransStart Tip Green qPCR SuperMix (Transgen Biotech, China) and GAPDH was performed as the endogenous control. The GAPDH primers were 5′-GAAGGTGAAGGTCGGAGTC-3′ and 5′-TCTACGAGTGGATGGTGCGTTG-3′. The PPM1E qPCR primers were 5′-GGTGCACCAAAGAAAGCAAA-3′ and 5′-CTCCCCTGTTGAACCCAAAT-3′. The Foxo3a qPCR primers were 5′-GGTGCACCAAAGAAAGCAAA-3′ and 5′-CTCTTGCCAGTTCCCTCATTCTG-3′. The GRP78 qPCR primers were 5′-CACAGTGGTGCCTACCAAGA-3′ and 5′-TGTCTTTTGTCAGGGGTCTTT-3′. The GRP94 qPCR primers were 5′-ACTCTAGGACGGGGAACGAC-3′ and 5′-CAGTTTCAGTCTTGCTGCTCC-3′. The ERP72 qPCR primers were 5′-CTACCCCACCATCAAGATCC-3′ and 5′-TGGTCAACACAAGCGTGACT-3′. The Chop qPCR primers were 5′-CCAAAATCAGAGCTGGAACC-3′ and 5′-CCATCTCTGCAGTTGGATCA-3′. The relative amount of mRNA compared to the internal control was calculated in the means of the 2^–ΔΔCT^.

### Western blot analysis

Protein was prepared with RIPA lysis buffer (150 mM NaCl; 0.5% sodium deoxycholate; 0.1% SDS; 1 mM EDTA; 50 mM Tris-HCl, pH 7.4; and 1 mM PMSF) and quantified by using a BCA kit (PC0020, Solarbio). Protein samples (15 μg) were separated by SDS-PAGE gel and then transferred to PVDF membranes (Bio-Rad). Then, the membranes were blocked in 5% fat-free milk at 25°C for 60 min, which were subsequently incubated with separate diluted primary antibody overnight at 4°C. And the associated primary antibodies were as follows: PPM1E (ab137122, Abcam), Myc (67447-1-Ig, Proteintech), Flag (66008-3-Ig, Proteintech), p53 (60283-2-Ig, Proteintech), p21 (60214-1-Ig, Proteintech) and AMPK-P (Thr172) (CY5608, Abways), PARP (13,371-1-AP, Proteintech), Cleaved-Caspase3 (9664S, CST), Caspase3 (19,677-1-AP, Proteintech), Caspase8 (13423-1-AP, Proteintech), Caspase9 (10380-1-A, Proteintech), Bax (50599-2-Ig, Proteintech), Cyto c (66264-1-Ig, Proteintech), Bim (22037-1-AP, Proteintech), Bcl-2 (12789-1-AP, Proteintech), Foxo3 (10849-1-AP, Proteintech), and Foxo4 (21,535-1-AP, Proteintech). The viral primary antibodies for Gag, Pol, Env, Bet, and Tas were all prepared in immunized rabbits in our laboratory. GAPDH (60004-1-Ig, Proteintech) and TBP (66166-1-Ig, Proteintech) were used as loading controls for separation for total or nuclear protein detection. After washing with TBST for three times, the membranes were incubated with horseradish peroxidase-conjugated IgG antibody at 25°C for 60 min. Exposure signal acquisition of tested protein was visualized by an enhanced chemiluminescence detection system (Tanon Corp., China).

### Luciferase reporter assay

After cell successful attachment, the PPM1E reporter gene plasmids and pCMV-Myc-PFV-Tas plasmids were transfected into HeLa cells. And luciferase assays were conducted by a luciferase reporter assay system (Promega, Madison, WI, USA) after transfection for 48 h according to the manufacturer’s instructions. Renilla luciferase activity was performed to normalize the transfection efficiency data. For the analysis of relative luciferase activity, it was calculated as the average of three independent experiments.

### Immunofluorescent assay

Immunofluorescence assay (IFA) was performed to identify the localization of HDAC4 (stained with rabbit anti-HDAC4 antibody (66838-1-Ig, Proteintech),and tetramethyl rhodamine isocyanate (TRITC)-conjugated goat anti-mouse secondary antibody(SA00013-3, Proteintech)), and 1 µg/ml Dox was added to overexpress Tas fusion and EGFP. HeLa-3 ˟Flag-Tas cells (3 ˟10^4^) were fixed with 4% paraformaldehyde at 25°C for 10 min, which was permeabilized with 0.1% Triton X-100 afterwhile in PBS at 25°C for 10 min. Furthermore, cells were blocked with 3% bovine serum albumin (BSA) in PBS and incubated with anti-Flag antibodies at 25°C for 2 hours. After incubating with rhodamine-conjugated secondary antibodies at 25°C for 45 min, the cells were fixed with 90% glycerol-PBS and examined with an Axio Imager Upright Microscope. Nuclei were stained with DAPI.

### Detection of ROS generation

The inducible expression cell lines were washed with PBS, treated with the fluorescent probe DHE (dihydroethidium) and incubated at 37°C for 60 min. Then, the cells were washed to detect ROS generation by flow cytometry. Finally, the inducible overexpression cell lines were treated with NAC (a ROS scavenger) at the concentrations of 0 mM, 0.08 mM, 0.4 mM, 2 mM, and 10 mM, and then, DHE assay and MTT assay were performed.

### Statistical analysis

All experiments were conducted independently 3 times, and the data were presented as the means ± SD (n = 3). A two-sample *t*-test was used to evaluate statistical significance and, compared to the control condition, the *p*-value was less than 0.05 indicated the consideration of statistically significant (*).

## Results

### Tas of foamy viruses inhibited cell proliferation

**Figure 1.**
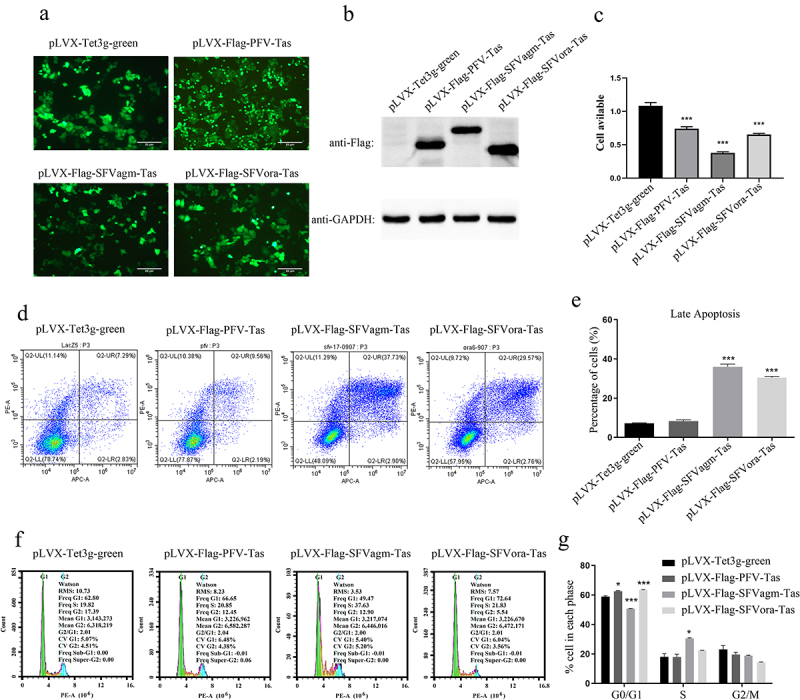
Tas of foamy viruses inhibited cell proliferation. The stable cell lines, pLVX-Flag-PFV-Tas, pLVX-Flag-SFVagm-Tas and pLVX-Flag-SFVora-Tas, were induced by 1ug/ml Dox for 48 h, and the stable cell line pLVX-Tet3g-green was used as a mock group. The expression of Tas was verified by fluorescence microscopy (a) and Western blotting (b), and the scale bars in images of microscope represent 20 µm. The cell proliferation was inhibited by SFVagm, SFVora and PFV Tas proteins with MTT assay (c). The apoptosis rate was confirmed by annexin V-APC and propidium iodide (PI) staining and confirmed by flow cytometry (d). The proportion of late apoptotic cells displayed that pLVX-Flag-SFVagm-Tas and pLVX-Flag-SFVora-Tas cell lines were induced significantly apoptotic compared to the mock group pLVX-Tet3g-green, but the pLVX-Flag-PFV-Tas cell line showed no difference. All data are presented as the mean ± SD (n = 3). *p < 0.05, **p < 0.01, ***p < 0.001 (e). The cell cycle arrest analysis was conducted by propidium iodide (PI) staining and confirmed by flow cytometry (f). The proportion of cells in different stages of division cycle was calculated by 3 independent experiments. pLVX-Flag-PFV-Tas and pLVX-Flag-SFVora-Tas cell lines were much more prompted in the G0/G1 phase, but pLVX-Flag-SFVagm-Tas cell line showed inverse results. All data are presented as the mean ± SD (n = 3). *p < 0.05, **p < 0.01, ***p < 0.001 (g).

To detect the importance of the transactivator Tas protein of the simian foamy virus, three stable HeLa cell lines, overexpressing Tas proteins of PFV, SFVagm, and SFVora, were generated and then the relative proliferation rates were measured by MTT assay (Figure 1(a,b)). Compared with the respective controls, Tas decreased the proliferation of HeLa cell lines, although the degree of influence on host cells differed (Figure 1(c)). As the transactivator Tas governs the level of viral transcript initiation, the differences in cell proliferation implied that foamy viruses might influence virus-host interactions by regulating different signaling pathways. The level of cell cytotoxicity was measured by annexin V-APC and propidium iodide (PI) staining, thereafter tested by flow cytometry. The rate of cell apoptosis indicated that Tas of SFVagm and SFVora induced significant apoptosis compared to the respective control group, but PFV Tas displayed no significant difference in apoptosis rate compared to the control group (Figure 1(d,e)). Furthermore, to evaluate the extent of growth inhibition by Tas, cell cycle analysis was also performed and the result suggested that Tas of PFV and SFVora significantly accumulated G0/G1 phase arrest, but SFVagm Tas caused many more cells to enter the S phase (figure 1(f,g)). These results display that Tas of PFV caused significant cell cycle arrest but not apoptosis, and Tas of SFVora caused both significant cell cycle arrest and apoptosis, whereas, for Tas of SFVagm, it induced the highest and most significant apoptosis rate and, as a consequence, a reversal in the cell cycle distribution results compared to those of PFV. Collectively, all three kinds of Tas inhibited cell proliferation.

### Tas of SFVagm and SFVora activated the mitochondrial apoptosis pathway by upregulating the expression of Foxo3a

**Figure 2.**
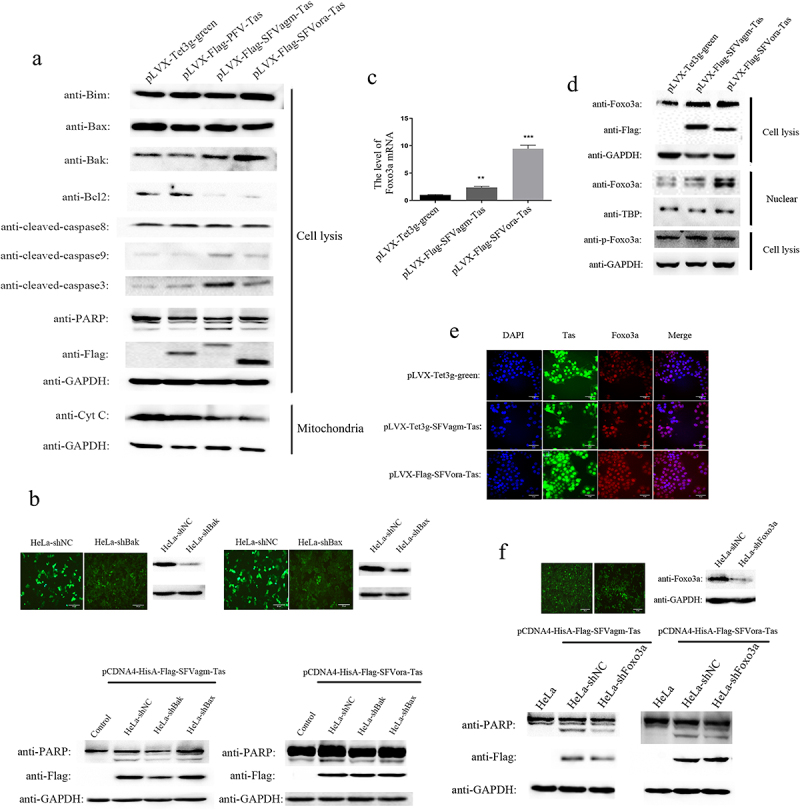
Tas of SFVagm and SFVora activated the mitochondrial apoptosis pathway by upregulating the expression of Foxo3a. The activation of mitochondrial apoptosis pathway-related proteins in SFVagm, SFVora and PFV Tas overexpressing stable cell lines was verified by WB (a). The stable cell lines HeLa-shBak and HeLa-shBax with mitochondria-associated protein Bak and Bax knockdown were verified and HeLa-shNC was used as a negative control (upper panel). Without Bak or Bax expression, the apoptosis induced by either SFVagm or SFVora were all rescued to varying degrees (lower panel), and the scale bars in images of microscope represent 20 µm (b). The expression of Foxo3a was increased by Tas of SFVagm or SFVora, as determined by RT-PCR (c). The regulation of SFVagm or SFVora Tas on the phosphorylation and nucleocytoplasmic distribution of Foxo3a was detected by WB. Although the total amount of Foxo3a increased, the expression level of phosphorylated Foxo3a did not change (d). The expression of Tas (green) in pLVX-Flag-SFVagm-Tas and pLVX-Flag-SFVora-Tas was induced by 1ug/ml Dox, and the expression of Foxo3a was detected by immunostaining with anti-Foxo3a (red). The cell nucleus was stained with DAPI (blue) and the merged data verified the nuclear transportation of Foxo3a, and the scale bars in images of microscope represent 10 µm (e). The stable cell line HeLa-shFoxo3a with Foxo3a knockdown was verified and HeLa-shNC was used as a mock group (upper panel). The cell apoptosis induced by Tas of SFVagm or SFVora was detected upon Foxo3a knockdown (lower panel). Without Foxo3a expression, the apoptosis induced by either SFVagm or SFVora were all rescued to varying degrees, and the scale bars in images of microscope represent 20 µm (f).

To investigate the exact mechanism of apoptosis induced by SFVagm and SFVora Tas, the expression of related proteins was evaluated. The results showed that the death receptor-mediated apoptotic factor cleaved caspase8 was not influenced but cleaved PARP, caspase3 and caspase9 were all significantly upregulated by Tas of SFVagm and SFVora. Moreover, compared to the proapoptotic factors Bim and Bax, which were almost impervious to SFVagm and SFVora Tas, the activation of another proapoptotic factor, Bak, and the inhibition of the antiapoptotic factor Bcl2 enhanced cytochrome c release (Figure 2(a)). Then, the proapoptotic factors Bak and Bax were knocked down to confirm that Tas of SFVagm and SFVora inhibited cell proliferation through mitochondrial pathway apoptosis. The results showed that without Bak and Bax, the expression of cleaved PARP was decreased, indicating that apoptosis was rescued (Figure 2(b)). Furthermore, through transcriptome sequencing, we discovered that Foxo3a, a major forkhead family of transcription factors protein, was significantly upregulated at the transcriptional level with SFVagm or SFVora Tas overexpression (Figure 2(c)). The increased Foxo3a expression induced by SFVagm and SFVora Tas was verified at the total protein level. As phosphorylation exerts great importance in the nuclear transfer shuttle and functional regulation of Foxo3a, the expression of p-Foxo3a was determined, and the results showed no difference in its expression (Figure 2(d)). However, it was confirmed that many Foxo3a proteins were transported from the cytoplasm to the nucleus upon Tas overexpression (Figure 2(d,e)). Combining all the results, the conclusion could be drawn that Foxo3a expression was promoted and Foxo3a was dephosphorylated by Tas of SFVagm and SFVora, which led to the nuclear transportation of Foxo3a. It has been extensively reported that Foxo3a is associated with the mitochondrial apoptosis pathway, and Foxo3a-knockdown cell lines have been constructed. When Foxo3a expression was abrogated, the level of cleaved PARP was decreased, which indicated that the apoptosis induced by SFVagm and SFVora Tas was rescued (figure 2(f)). All these results suggested that Tas of SFVagm and SFVora activated the expression and nuclear transport of Foxo3a, which activated the mitochondria-mediated apoptosis pathway to inhibit cell proliferation.

### Tas of SFVagm and SFVora increased the expression of Foxo3a by activating ROS generation and ERS

**Figure 3.**
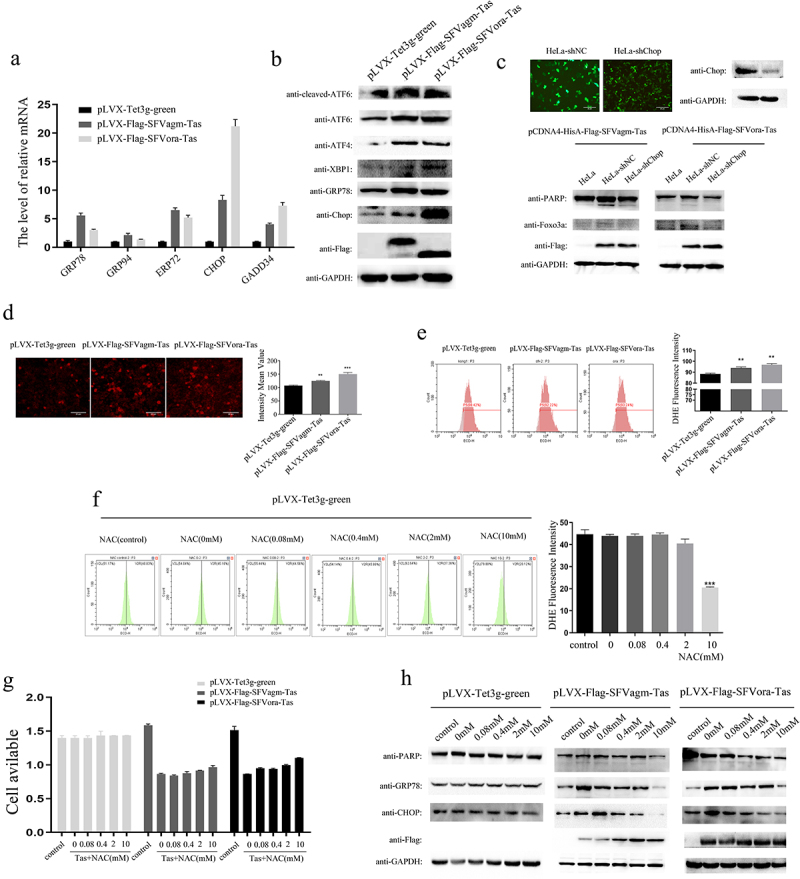
Tas of SFVagm and SFVora increased the expression of Foxo3a by activating ROS generation and ERS. Endoplasmic reticulum stress (ER stress) pathway-related proteins were activated in SFVagm and SFVora Tas-overexpressing stable cell lines as determined by RT-PCR (a) and WB analysis (b). The stable cell line HeLa-shChop with Chop knockdown was verified and HeLa-shNC was used as a mock group (upper panel). Without Chop expression, the apoptosis and Foxo3a expression induced by either SFVagm or SFVora Tas were all rescued to varying degrees (lower panel), and the scale bars in images of microscope represent 20 µm (c). The DHE (dihydroethidium) assay of ROS generation increased by Tas of SFVagm or SFVora was detected by fluorescence microscopy (left) and the density statistics were calculated (right), and the scale bars in images of microscope represent 20 µm. All data are presented as the mean ± SD (n = 3). *p < 0.05, **p < 0.01, ***p < 0.001 (d). The DHE (dihydroethidium) assay of ROS generation induced by Tas of SFVagm or SFVora was confirmed by flow cytometry (left) and the density statistics were calculated (right). All data are presented as the mean ± SD (n = 3). *p < 0.05, **p < 0.01, ***p < 0.001 (e). The impact of N-Acetyl-L-cysteine (NAC, used for ROS elimination) of different concentration gradients (0 mM, 0.08 mM, 0.4 mM, 2 mM, and 10 mM) on ROS generation was tested by flow cytometry (left) and the density statistics were calculated (right) to show the decrement of ROS induced by NAC. All data are presented as the mean ± SD (n = 3). *p < 0.05, **p < 0.01, ***p < 0.001 (f). The cell proliferation inhibition effect of NAC of different concentration gradients (0 mM, 0.08 mM, 0.4 mM, 2 mM, and 10 mM) on the SFVagm or SFVora Tas-overexpressing stable cell lines was verified by MTT assays (g). The cell apoptosis and ER stress regulation induced by Tas of SFVagm or SFVora was rescued with the increasing of NAC concentrations (h).

To explore the mechanism of SFVagm and SFVora Tas on the activation of Foxo3a, the detection of molecular chaperone proteins that bind to improperly folded proteins, which marks the initiation of endoplasmic reticulum stress, was performed, and the transcripts of GRP78, GRP94, ERP72, Chop, and GADD34 were all found to be upregulated (Figure 3(a)). Moreover, the increase in ATF6, ATF4, and XBP1 expression at the translational level confirmed the initiation of ERS (Figure 3(b)). Furthermore, in stable cell lines in which Chop, a typical protein in the ERS pathway, was knocked down, the activation of Foxo3a induced by either SFVagm Tas or SFVora Tas was significantly attenuated, and apoptosis was inhibited (Figure 3(c)). All these results suggested that Tas of SFVagm and SFVora upregulated Foxo3a expression and induced apoptosis via ERS initiation. Furthermore, another important event, the release of ROS, which induces ERS, was also assessed. DHE probe staining and flow cytometry were used for detecting the concentration of ROS, and the result showed that Tas of SFVagm and SFVora enhanced ROS production (Figure 3(d,e)). Then, NAC was used to enhance the removal of ROS, and the result suggested that with increasing NAC concentration, ROS levels were dramatically decreased (figure 3(f)), and the inhibition effect on cell proliferation induced by SFVagm and SFVora Tas was weakened (Figure 3(g)). In addition, ERS-related proteins and the apoptosis-related protein were found to be decreased in a concentration-dependent manner with increasing NAC concentration (Figure 3(h)). These results showed that Tas of SFVagm and SFVora increased the expression of Foxo3a via the induction of ERS by ROS that had been generated at a high rate.

### Tas of PFV promoted cell cycle arrest through PPM1E upregulation and the nuclear transfer shuttle of HDAC4

**Figure 4.**
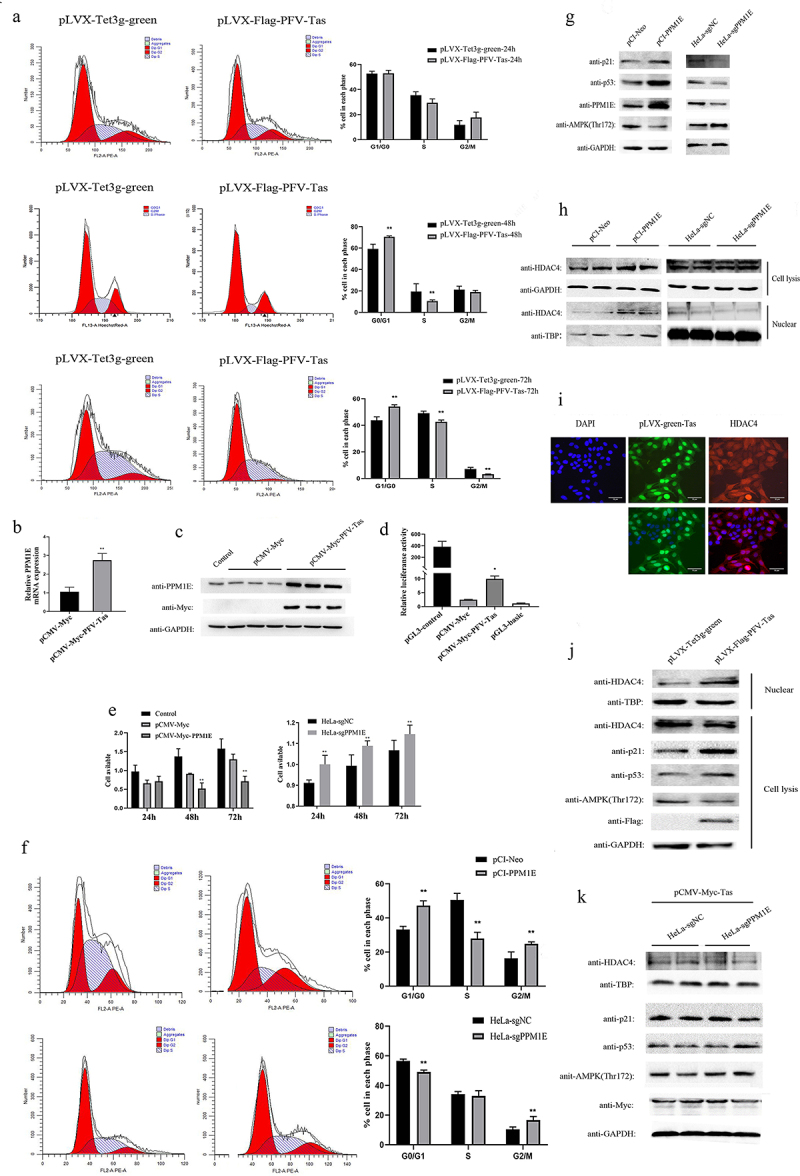
Tas of PFV promoted cell cycle arrest through PPM1E upregulation and the nuclear transfer shuttling of HDAC4. Cell cycle arrest analysis with PFV Tas overexpressed at 24 h, 48 h and 72 h separately were performed with propidium iodide (PI) staining and confirmed by flow cytometry (left), and the histograms displayed much more G0/G1 phase distribution induced by PFV Tas. All data are presented as the mean ± SD (n = 3). *p < 0.05, **p < 0.01, ***p < 0.001 (a). Upregulated of PPM1E by PFV Tas was verified by RT-PCR (b) and WB analysis (c). The activation of PFV Tas on PPM1E promoter was verified by luciferase reporter assay, pGL3-control and pGL3-basic was conducted as the positive or negative control, respectively (d). MTT assay was used to test the impact of either overexpression (upper panel) or knockout (lower panel) of PPM1E on cell proliferation. The histograms displayed that overexpression of PPM1E inhibit cell proliferation whereas PPM1E knockout promote cell proliferation. All data are presented as the mean ± SD (n = 3). *p < 0.05, **p < 0.01, ***p < 0.001 (e). Cell cycle arrest was analyzed with PPM1E overexpression or PPM1E knockout by flow cytometry (left) and the histograms displayed that overexpression of PPM1E promoted G0/G1 phase cell arrest whereas PPM1E knockdown showed less cells arrested in G0/G1 phase. All data are presented as the mean ± SD (n = 3). *p < 0.05, **p < 0.01, ***p < 0.001 (f). The dephosphorylation on AMPK and the upregulation on p53-p21expression by PPM1E were verified by WB (g). The total expression of HDAC4 was not influenced by PPM1E, whereas the nuclear expression of HDAC4 was upregulated by PPM1E (h). The expression of Tas (green) in pLVX-Flag-PFV-Tas cell lines were induced by 1ug/ml Dox, and the expression of HDAC4 was detected by immunostaining with anti-HDAC4 (red). The cell nucleus was stained with DAPI (blue) and the merged data verified the nuclear transportation of HDAC4, and the scale bars in images of microscope represent 10 µm (i). The regulatory effect of PFV Tas on the phosphorylation of AMPK, the regulation of p53-p21 expression and the nuclear transportation of HDAC4 was determined with PPM1E (j) or without PPM1E (k).

In contrast to the apoptosis induced by Tas of SFVagm and SFVora, overexpression of PFV Tas led to the G0/G1 phase cycle arrest; this finding was confirmed by flow cytometry at different temporal stages. The results showed that in contrast to the nonsignificant difference compared with the control group at 24 h, 22.26%, and 26.33% of the cells were driven into the G0/G1 phase at 48 h and 72 h, respectively (Figure 4(a)). According to transcriptome sequencing, *ppm1e*, a dephosphorylase gene, was clearly upregulated at the transcriptional level (Figure 4(b)), and the translational upregulation of PPM1E was also verified (Figure 4(c)). Furthermore, the inhibition of cell proliferation with increased PPM1E overexpression and, reciprocally, the promotion of cell proliferation upon PPM1E knockout were confirmed by MTT assay (Figure 4(e)). Then, overexpression of PPM1E was shown to facilitate G0/G1 phase arrest in cells, which was verified by experiments in which PPM1E was knocked out (figure 4(f)). To explore the mechanism by which PPM1E inhibits cell proliferation, p53 and p21 were found to be upregulated, and AMPK was found to be dephosphorylated at Thr172 (Figure 4(g)). Experiments with the cell cycle progression regulator HDAC4 verified that, although the total protein level of HDAC4 did not change, nucleoprotein expression was significantly upregulated (Figure 4(h)), which was proven by immunofluorescence colocalization of PFV Tas and HDAC4 (Figure 4(i)). Similar tests were performed with the PFV Tas-overexpressing group cells, and the results indicated that p53, p21, and nuclear-localized HDAC4 were all upregulated and that AMPK-Thr172 was dephosphorylated (Figure 4(j)). To further prove that PFV Tas regulates downstream factors that are important for cell cycle arrest, PPM1E was knocked out by the lentiCRISPRv2 system, and the results showed that PFV Tas exerted no effect without PPM1E expression. Therefore, we concluded that PFV Tas induced the activation of p53-p21 and AMPK-HDAC4 signaling pathway by increasing PPM1E expression, which finally contributed to cell cycle arrest.

## Discussion

Persistent and chronic infections of PFV without pathogenicity appear either naturally or accidentally in infected hosts *in vivo*. The reasons for the nonpathogenic association of foamy virus have been mainly attributed to the following viral characteristics: 1) preferential integration into transcriptionally inactive regions of host cells resulted in an apparent lack of association with clinical disease [37]; 2), the unique self-replication mechanism prevented foamy viruses from acquiring many RT-induced mutations over a short time by leveraging the complex repair systems of host cells [38]; 3), FV replication is limited to epithelial cells in a very late stage of differentiation, immediately prior to cell shedding from the tissue. These mechanisms of viral replication promote efficient virus transmission via shedding cells while limiting viral replication at a superficial site, minimizing host tissue damage. Indeed, in the majority of infections, none of the foamy viruses cause pathogenic sequelae, underlining a homeostatic mechanism of foamy virus infection in host mammals that coevolved with the virus and host over a long period [4]. Although infection is limited to superficial oral mucosal epithelial cells *in vivo* and there is no appropriate tissue culture system for foamy virus infection *in vitro*. And Foamy virus has been found to infect a broad range of permissive host cell types. For the infection on fibroblasts, epithelial cells, and neural cells, they all exhibit severe cytopathic effects, which result in syncytial body formation and foam-like vacuolation and, finally, death. However, there is no cytopathology associated in T cell-derived Jurkat cells or Hut-78 cells. And for the infections on B- and macrophage-originated cells, the cytopathic effects are delayed by several days [39]. These findings are explained by the ratio of two nonstructural proteins, Tas and Bet, which regulate foamy virus replication in latent and persistent infections [40]. As an important initiation transactivator, PFV Tas can dramatically increase the transcription of *p57Kip2, IGF-II*, and *EphB3*, thereby regulating host cell processing during the process of viral infection [41].

**Figure 5.**
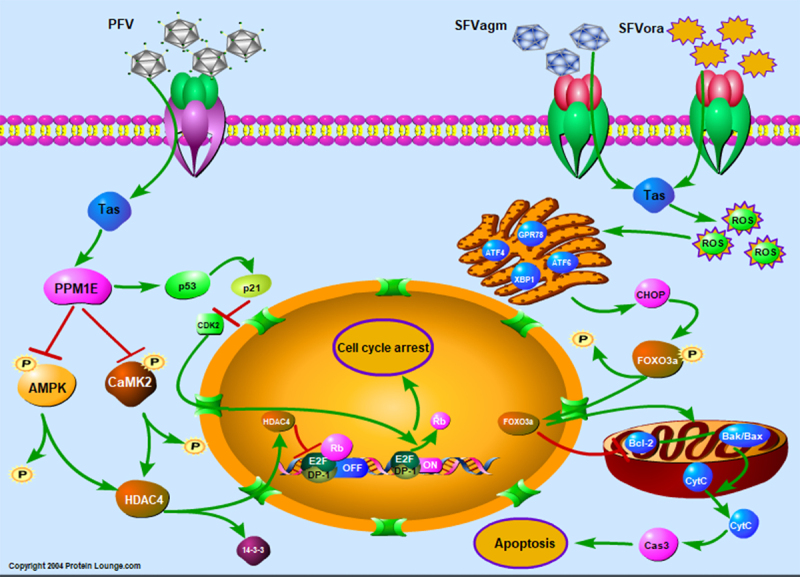
Diagram of the cell proliferation mechanism induced by different foamy virus Tas proteins through cell cycle arrest or apoptosis. For the inhibition of cell proliferation induced by SFVagm or SFVora Tas protein, Foxo3a is upregulated via generation of ROS and initiation of ERS, and which ultimately accounts for the activation of the mitochondrial apoptosis pathway. PFV Tas protein promotes G0/G1 phase arrest through the p53-p21 signaling pathway and AMPK-HDAC4 signaling pathway, which are induced by the upregulated PPM1E.

In this study, we confirmed that three kinds of foamy virus transactivator Tas can inhibit cell proliferation, implying that early regulatory events are triggered upon viral infection. Furthermore, we found that Tas of SFVagm and SFVora, which were isolated from the host of OWM, led to notable apoptosis. A previous study indicated that SFVagm infection induced the formation of apoptotic bodies, triggered the condensation of nuclear chromatin and then led to apoptotic cell death [42]. However, the exact molecular mechanism of this phenomenon remains unknown. In contrast to HTLV-1, which is also transmitted from simians to humans and leads to adult T-cell leukemia/lymphoma (ATLL), the transactivator Tax was reported to protect cells from apoptosis by regulating mitochondrial apoptosis pathway-associated cytokines expression, such as Bcl-2, Bcl-XL [43–45] and Bak, Bax [46]. Other reports indicated that downregulation of the apoptotic regulators p53 [47,48], caspase3 and caspase8 [49] by Tax may account for the protection of cells from apoptosis. Tat, the transactivator of HIV, was shown to induce cell apoptosis mediated by activating NF-kB signal pathway and the increasing the generation of ROS [50–52]. Besides, Tat was also shown to initiate ERS by increasing the expression of ATF6, PERK, IRE1 and Chop [53], which leads to the activation of caspase12 and caspase3 [54]. Furthermore, some studies have also suggested that Tat can promote nuclear transfer shuttle of Foxo3a by upregulating the PTEN expression and enhancing the Foxo3a dephosphorylation, which ultimately leads to the regulation of Bcl-2 and Bim and then to apoptosis [55,56]. Thus, we confirmed that Tas of SFVagm and SFVora can upregulate the expression of Foxo3a induced via the upregulating on ROS generation and the ER stress activation, which results in mitochondrial apoptosis ultimately (Figure 5).

On the other hand, it was found that PFV Tas facilitated significant G0/G1 accumulation, an effect quite different from that of SFVagm or SFVora Tas. The interaction of viruses and cytokines was reported to interfere with nucleolar function and result in cell cycle arrest [57,58]. Recent studies revealed that in the process of hepatocellular carcinoma development caused by HBV infection, the virus regulates host cell cycle arrest by phosphorylating the cytokine 4E-BP1 [25], which implied that phosphorylation is of great importance in cell cycle arrest induced by virus infection. PPM1E, which belongs to the Ser/Thr protein phosphatase PPM family, was found to regulate signal transduction and cell proliferation [59]. PPM1E can interact with AMPK and dephosphorylate it at Thr172, leading to the inactivation of AMPK [31,32]. Another important dephosphorylated substrate of PPM1E is the Ca2+/calmodulin-dependent protein kinase (CaMK) family [60]. The phosphorylated AMPK and CaMKII may increase the phosphorylation of another histone deacetylase family (HDACs), which results in the transport of HDACs out of the nucleus with the action of the molecular chaperone 14-3-3. Then, it makes for the transcription of downstream genes that are critical in transcriptional regulation and cell cycle progression [35,61–63]. In our study, PPM1E, increased by PFV Tas dramatically, not only dephosphorylated AMPK but also dephosphorylated CaMKII, and this led to the nuclear transfer shuttle of HDAC4 but did not affect its expression. Interestingly, as an important regulatory cytokine, which has been suggested to account for cell cycle arrest, apoptosis, and senescence, or respond to diverse cellular stresses, such as DNA damages and changes in metabolism, p53 was also verified to be the downstream factor of PPM1E in our study [64]. Recent studies have revealed that PPM1F can dephosphorylate p53 at Ser20 and lead to cell cycle arrest [65]. However, in our study, we demonstrated that PPM1E increased the activation of the p53 signaling pathway. Therefore, it could be concluded that Tas of PFV upregulates PPM1E, which dephosphorylates AMPK and CaMKII. Finally, the abrogated effect of inactivated AMPK and CaMKII on HDAC4 accounts for the nuclear transportation of HDAC4 and the inhibition of the transcriptional regulatory complex. Furthermore, PPM1E promotes the expression of p53 and the inhibition of CDK2, which leads to the G0/G1 phase arrest (Figure 5).

In conclusion, we demonstrated that foamy viruses inhibit cell proliferation via their transactivator Tas through apoptosis or cell cycle arrest. Moreover, an interesting question emerged: Why do the Tas proteins of different foamy viruses lead to significantly different responses in host cells? The molecular mechanism that explains this difference needs go into more depth about the adaptation of the proteins structure to function. Collectively, these findings imply that different mechanisms are involved in virus-host interactions and reveal new horizons in the resistance of host cells to foreign invasion by viruses and the survival strategies of these viruses.

## Data Availability

Data sharing is not applicable to this article as no new data were created or analyzed in this study.
